# The Frequency of Use and Harm Perception of Heated Tobacco Products (HTPs): The 2019 Cross-Sectional Survey among Medical Students from Poland

**DOI:** 10.3390/ijerph18073381

**Published:** 2021-03-24

**Authors:** Paulina Majek, Mateusz Jankowski, Bartłomiej Nowak, Maksymilian Macherski, Maciej Nowak, Aleksandra Gil, Piotr Nakiela, Barbara Lewicka, Joshua Allan Lawson, Jan Eugeniusz Zejda, Grzegorz Marek Brożek

**Affiliations:** 1 Department of Epidemiology, School of Medicine in Katowice, Medical University of Silesia, 40-752 Katowice, Poland; b.n.3@interia.eu (B.N.); maksm123@gmail.com (M.M.); maciejnowak11111@gmail.com (M.N.); aleksandra.gil97@gmail.com (A.G.); piotr.nakiela@med.sum.edu.pl (P.N.); barbara.e.lewicka@gmail.com (B.L.); jzejda@sum.edu.pl (J.E.Z.); gbrozek@sum.edu.pl (G.M.B.); 2 School of Public Health, Centre of Postgraduate Medical Education, 01-826 Warsaw, Poland; mjankowski@cmkp.edu.pl; 3 Canadian Centre for Health and Safety in Agriculture, College of Medicine, University of Saskatchewan, Saskatoon, SK S7N 2Z4, Canada; josh.lawson@usask.ca; 4 Department of Medicine, College of Medicine, University of Saskatchewan, Saskatoon, SK S7N 0W8, Canada

**Keywords:** heated tobacco products, heating tobacco, smoking, tobacco, students

## Abstract

Heated tobacco products (HTPs) are devices for generating a nicotine aerosol by heating the tobacco sticks. This study aimed to assess (1) the prevalence of HTP and tobacco cigarette usage among medical students, (2) to characterize smoking habits and (3) to assess students’ awareness and opinions about HTPs. A cross-sectional survey on the frequency and attitudes toward cigarettes, e-cigarettes and HTP use was performed between 2019–2020 at the Medical University of Silesia in Katowice (Poland). The data were obtained from 1344 students aged 21.8 ± 1.9 years (response rate: 66.9%). Current traditional tobacco use was 13.2%, e-cigarettes use 3.5%, and HTP use 2.8% of students. Duration of use was shorter among HTPs users comparing to cigarette smokers (*p* < 0.001) although the number of tobacco sticks used daily was similar (*p* = 0.1). Almost 30% of respondents have ever tried HTPs. HTPs were considered safe by 5.3% of respondents (43.2% of HTP users vs. 3.9% of non-HTP users, *p* < 0.001). HTP users were more likely to report that heating tobacco is not addictive (odds ratio (OR) = 8.9, 95% confidence interval (CI): 1.8–45.8) and disagreed with a public ban on HTP use (OR = 4.9, 95%CI: 2.5–9.8). Among students, HTP use was less popular than cigarette smoking, but awareness of their presence is widespread.

## 1. Introduction

According to the World Health Organization (WHO) data, in 2015, one quarter (24.9%) of the world’s adult population were current users of some form of tobacco [1]. Similar data were obtained in the Special Eurobarometer 458 survey in 2017, where over a quarter (26%) of European Union (EU) citizens were cigarette smokers [2]. Poland is over the European average at 30% and ranks sixth out of the 28 countries based on frequency of cigarette smoking [2]. In recent years, new forms of nicotine-containing products, such as electronic cigarettes (e-cigarettes) and heated tobacco products (HTPs), have been gaining popularity [3].

HTPs were introduced for the first time in 1988, in the USA, however, this technology initially did not gain wide popularity and was discontinued shortly after its introduction. Each reincarnation of HTPs was commercially unsuccessful [4]. Recently, the tobacco industry has made another attempt to introduce HTPs to the market. In April 2017, the newest HTPs for the first time were introduced to the Polish market, just 3 years after their worldwide premiere. The main principle of HTPs’ functionality is based on a heating tobacco stick that leads to the creation of aerosol containing nicotine [5,6]. Heating technology heats the tobacco to significantly lower temperatures (up to 350 °C) compared to traditional cigarettes (approximately 500 °C). Presently, two types of HTP are available in Poland—IQOS made by Philip Morris International and glo produced by British American Tobacco. The technology of IQOS allows for a maximum of 14 puffs or 6 min of using whilst consuming a single tobacco stick applied on a heating blade [3,5]. Glo consists of the heating tube where the tobacco rod is heated from the periphery. The product reaches operating temperature after approximately 30–40 s, and each heating session lasts for an additional 3 min [7]. Unlike e-cigarettes, tobacco sticks used in HTPs are made of tobacco soaked in propylene glycol [8]. Length of HTPs use, appearance of these sticks, and their nicotine level may contribute to smoking behaviors similar to those of traditional cigarette use.

E-cigarette use is most popular among teenagers and young adults. In Poland, the overall frequency of e-cigarette use among university students was 2.9%, which is higher than that observed in the general population (1%) [2,3]. Moreover, there are geographical differences in the frequency of e-cigarette use across Poland [9]. There is a lack of epidemiological research focused on the frequency of HTPs, especially among the young adults. Data from Italy and Japan, where these products were tested for the first time in 2014, indicate a steadily increasing frequency of HTPs use [5,10]. The 2019 national survey in Poland revealed the popularity of HTPs among adults was 0.4% use, but there is no data about the frequency of HTP usage among young people [11]. Thus, we began this assessment among medical students, as this is a group of young adults who are, on average, 22 years old. This would help provide some evidence about HTP use in younger adults in Poland. An additional advantage is that they are a group with whom we have worked before [12]. Given the growing popularity of these devices and their style of promotion, where they are shown as trendy, it is possible that HTP use frequency among young adults is higher than in the general population, as well as is the case of e-cigarettes [2,3].

The objectives of this study were to assess (1) the prevalence of HTP and tobacco cigarette usage among medical students, (2) to characterize smoking habits and (3) to assess students’ awareness and opinions about HTPs among medical students of the Faculty of Medical Sciences at the Medical University of Silesia in Katowice (Poland). 

This study is a third wave (of cross-sectional surveys) carried out among medical students at the Medical University of Silesia in Katowice (2016–2017/2018–2019/2020) [3,12]. This was the first time HTPs were included in the survey.

## 2. Materials and Methods

### 2.1. Study Design and Population 

A survey was conducted between October 2019 and February 2020 at the Faculty of Medical Sciences at the Medical University of Silesia in Katowice (Poland). The student population was 2370 at this time. Students from years 1–5 attending University were eligible to be included in the research (*n* = 2008). The sixth-year students were not invited, due to the fact that contact classes were cancelled when the authors planned to survey them (as a consequence of the government’s decision in response to the COVID pandemic). All questionnaires were printed and delivered to each of the subjects personally by a member of the research team. Questionnaires were answered by students during breaks between classes or lectures. Participation in the study was voluntary and anonymous. Participants had the right to refuse to participate without giving a reason. The study protocol was reviewed and approved by the Ethical Review Board at the Medical University of Silesia, Poland (decision number: PCN/0022/KB1/59/I/19). The questionnaire was anonymous, so it was not possible to agree to participate in writing. The letter of invitation contained information that the fulfilment and return of the questionnaire implied an implicit consent to participate in the survey.

### 2.2. Study Questionnaire

The research tool was an original questionnaire developed for the purpose of this study (Supplemental File–Study Questionnaire). The questionnaire included 18 questions related to the awareness and attitude towards the use of HTPs, e-cigarettes and traditional cigarettes as well as their frequency of use. The questionnaire was based on previous research projects on similar subjects and specifically on the questionnaire used in YoUng People E-Smoking Study (YUPESS) used by our team [3]. The questions also addressed safety concerns (health impact), smoking in public places, and views toward the legislation of HTPs. As the term “heating tobacco products” is not used commonly among the consumers, we clarified it in the introduction to the questionnaire: “Heated Tobacco Products (HTPs) as “IQOS” or “glo” are innovative tobacco products, which are advertised as alternative for traditional smoking”. Heated tobacco product awareness was defined according to answers of the following questions: “Have you ever heard of heated tobacco products?” (“Yes”/“No”). Questions to determine if a student had ever used a cigarette, e-cigarette, or heated tobacco products included “Have you ever smoked/tried a combustible cigarette?”, “Have you ever used/tried an e-cigarette (even one puff)?” and “Have you ever used/tried heated tobacco products?” Current smoking status was based on the questions: “Do you currently smoke combustible cigarettes?”, “Do you currently use e-cigarettes?” and “Do you currently use heated tobacco products?”, each with two possible answers (“Yes” or “No”) with the possibility of completing “How long have you been using cigarettes/e-cigarettes/HTPs?” (“number of months”) and “How many cigarettes/tobacco sticks do you smoke/use on average per day/how many times per day do you use an e-cigarette?” (“number/day”). Moreover, questions regarding harm perception were also addressed, and were based on the questions: “Do you think Heated Tobacco Products are safe for your health/passive smokers/pregnant women?”, “Do you think you can become addicted to Heated Tobacco Products?”, “In your opinion, do you think that using heated tobacco products in public places should be banned?” (“Yes/No/No opinion”).

The questionnaire reliability was checked in a pilot study where 37 students completed the identical survey twice, 7–14 days apart. Depending on the question and subscale, the kappa coefficient ranged from 0.64 to 1.0 for the most critical questions.

Subjects were classified into one of five groups based on their current smoking status: HTP users only, traditional cigarette smokers only, e-cigarette smokers only, dual users (people who use traditional cigarettes and e-cigarettes or HTPs) and non-users to assess the frequency of smoking and estimate the pattern of smoking among tobacco users. To analyze opinions about safety, possibility of addiction, legalization of usage in public spaces and promotion of HTPs, respondents were divided into two groups—HTPs users, and non-HTPs users. Students who have never heard about HTPs were excluded from the analysis about the opinions.

### 2.3. Statistical Analysis 

The data was analyzed with Statistica 13.3 Software (TIBCO Software Inc., Palo Alto, CA, USA) and IBM SPSS Statistics, version 26 (IBM, Armonk, NY, USA). Normality of distributions of continuous variables was assessed by the Shapiro–Wilk test. Statistical significance of differences between continuous variables was analyzed by the independent samples *t*-test or the Mann–Whitney U test if the assumptions for the *t*-test were not met. Distribution of categorical variables was shown by frequencies and proportions along with 95% confidence intervals. Statistical testing to compare between categorical variables was completed using the independent samples Chi-square test or the Fishers Exact Test is warranted when the sample size is insufficient for the Chi-square test (the assumption is when less than 20% of cells have an expected cell count of <5). Statistical inference was based on the criterion *p* < 0.05. To control for potential confounds, we used multiple logistic regression where the strength of associations was determined by the odds ratio and 95% confidence intervals (CIs).

## 3. Results

Data were obtained from 1344 medical students (response rate: 66.9%). The average age of the respondents was 21.8 ± 1.9 (range 18–33), with no difference by sex (*p* = 0.1). The group consisted of 839 women (62.4%) and 505 men (37.6%) which reflects the sex distribution of the students of medicine at the University. Most (76.5%) students have heard about heated tobacco products—mainly from family and friends (74.6%), shop windows (47.6%) and advertising materials (35.6%).

Almost 30% of respondents have tried HTPs. The mean age of nicotine initiation differed among the types of nicotine-containing products including traditional cigarettes (16.5 ± 2.5 years), e-cigarettes (18.0 ± 2.2) and HTPs (20.9 ± 1.9). Students had tried HTPs at an older age, in comparison to traditional cigarettes and e-cigarettes (*p* < 0.001), as well as between e-cigarettes and HTPs (*p* < 0.001).

### 3.1. Frequency of Smoking, E-Smoking and Heated Tobacco Product (HTP) Usage

Overall, 13.2% of participants currently smoked traditional cigarettes, 3.5% currently used e-cigarettes, and 2.8% currently used HTPs. Non-user status was declared by 83.0% students. In a group of HTPs users, almost half of them also used other types of cigarettes. The prevalence of HTP use was 4.0% among men and 2.0% among women. The proportion of students by smoking status and sex is shown in the Table 1, with division into five specific groups: HTP users only, traditional cigarette smokers only, e-cigarette smokers only, dual users (people who use traditional cigarettes and e-cigarettes or HTPs) and non-users. Small differences in percentages in text and Table 1 arise from excluding from the table singular cases where respondents use simultaneously HTPs and e-cigarettes (*n* = 1), or HTPs, e-cigarettes and traditional cigarettes (*n* = 3).

### 3.2. Pattern of Use

The duration (25th to 75th percentile) of smoking among exclusive traditional smokers was 24 (7–48) months while it was 12 (6–24) months among e-cigarette users and 6 (4–11) months among HTP users. The duration of smoking was shorter among users of HTPs compared to cigarette smokers (*p* < 0.001, Mann-Whitney U test). Among exclusive HTP users (*n* = 17) the duration of usage was less than or equal to 6 months for 52.9% of students while 23.5% smoked between 6 and 12 months, 23.5% of persons smoked between 1 and 5 years, and no-one smoked for over 5 years. Among cigarette smokers (*n* = 139) the respective proportion of smokers were 23.0%, 10.1%, 58.3% and 8.6%.

On average, traditional smokers smoked 4 (2–8) cigarettes, e-cigarette users had 10 (3–20) sessions of smoking, and HTP users vape 7.1 ± 4.4 tobacco sticks daily. HTP users were likely to smoke a similar number of tobacco sticks per day compared to the number of cigarettes smoked by traditional smokers (*p* = 0.2—Mann-Whitney U test). Up to 5 cigarettes per day were smoked by 66.7% of cigarette smokers and 38.9% of HTP users; smoking 6–15 cigarettes per day was declared by 29.8% cigarette smokers and 61.1% of HTP users; smoking over 15 cigarettes per day was declared by 3.6% of cigarette smokers. Pattern of nicotine containing product use is shown in the Table 2.

Among 18 non-exclusive HTP users, which means those who use simultaneously other types of nicotine containing products, the duration of using HTPs (4 (1–11) months) was significantly shorter (*p* < 0.001, Mann–Whitney U-test). compared with tobacco smoking (40, (15–60) months) but they smoked a similar number of cigarettes and tobacco sticks daily (8.1 ± 6.1 vs. 7.3 ± 6.5; *p* = 0.6, Mann–Whitney U-test). Furthermore, the average number of tobacco sticks used daily by exclusive HTP users and remaining HTP users did not differ statistically (7.1 ± 4.4 vs. 7.3 ± 6.5; *p* = 0.8, Mann–Whitney U test). Half of them used HTP less than 6 months, and only 6% more than 1 year, while more than the half smoked traditional cigarettes longer than 1 year (almost 40% 1–5 years, and almost 20% more than 5 years).

### 3.3. Opinions about Safety of HTPs

Respondents, who have never heard about HTPs were excluded from this analysis. Only 5.3% of students declared that HTPs are safe for health. This opinion differs according to smoking status. Among HTP users, 43.2% believe they are safe in comparison to 3.9% of non-HTP users (*p* < 0.001). Also, 20.4% of students believe they are safe for passive smokers, defined as people who inhale HTP aerosol while standing next to users. Approximately half of the respondents believed they are safer than traditional cigarettes and almost 1/4 believed they are safer than e-cigarettes. The belief that HTP use can lead to addiction was shared by 93.9% of respondents and over 80% claimed that they were as addictive as traditional cigarettes. Most respondents (74.1%) believed that HTPs should be forbidden in public places. However, only 38.9% of HTPs users support vaping prohibition in public places, compared with 75.3% of those who do not use HTPs (*p* < 0.001). The findings about perception of HTPs are shown in the Table 3.

### 3.4. Promotion of HTPs

Among respondents who have ever heard about HTPs more than 90% have encountered any type of promotion and marketing of heated tobacco. The most common types of promotion were: point-of-sale advertising—stores/kiosks etc. (81.5%), Internet advertising (62.2%), booths of tobacco companies during mass events, e.g., concerts (46.9%), sale of heated tobacco at a promotional price (37.8%). There were differences in exposure relative to different promotions between those who use HTPs and those who do not. HTP users more often than non-HTP users experienced a promotion such as the sale of heated tobacco at a promotional price (86.5% vs. 36.0%; *p* < 0.001), free trial at the point of sale (78.4% vs. 27.6%, *p* < 0.001), booths of tobacco companies during mass events (73.0% vs. 45.9%; *p* < 0.001), sponsoring of cultural/sporting events by tobacco companies (62.2% vs. 28.5%; *p* < 0.001) and articles with the logo/name (46.0% vs. 19.6%; *p* < 0.001). There were no differences in numbers for point-of-sale advertising and Internet advertising.

### 3.5. Adjusted Analysis

Results from the adjusted analysis (Table 4 and Table 5) confirmed the previous results. Males were more likely to try e-cigarettes while students from the later years were less likely to try them. Older students were more likely to have tried traditional cigarettes and be current cigarette smokers. Males compared to females were also more likely to be current e-cigarette smokers and HTP users.

Results from analyses examining the perceptions of HTP usage are presented in Table 5. According to the safety of HTPs for users, for passive smokers and also for pregnant women, HTP users were less likely to think it was not safe. HTP users were more likely to believe that heating tobacco is not addictive and to not agree with a ban on public use of HTPs.

## 4. Discussion

The results of our study showed high awareness about HTPs. Our findings revealed that men are at a greater risk of smoking and using HTP. It is worrying that most HTP users are multiple users, indicating that the use of HTPs is to be considered more as an additional route of nicotine delivery than a fulfilling product. By contrast with marketing strategies presented by the tobacco industry, most of our respondents do not believe in HTP safety and HTP users were less likely to believe that heating tobacco is as addictive as traditional cigarettes. 

### 4.1. Knowledge Concerning HTPs

The social awareness regarding the existence and impact of HTPs is increasing. In contrast to our results, a study conducted by Italian researchers has shown that in 2017 almost ¾ of respondents in the age group 15–24 years have never heard of HTPs [13]. What is more, an online survey carried out the same year among young Koreans showed a similar relationship—only 38.1% of participants were aware of HTPs [14]. Furthermore, HTP awareness is highest among younger adults and among men [15,16]. Overall, the number of people who have used and heard of HTPs is constantly increasing [5,15]. The interest and the willingness to use HTPs is significantly higher amongst current cigarette smokers who intend to quit smoking compared to cigarette smokers who do not intend to quit [5,10]. That suggests the use of HTPs as a gateway to quit smoking. However, there is an absence of studies assessing the potential role of HTPs as a tool to quit smoking. 

The promotion of HTPs is primarily focused on young people [17,18,19]. Advertisement of the HTPs is widespread, especially on the Internet, and social media platforms, such as Instagram, where communities centered around HTP use have been created [19,20]. An analysis of the behavior of HTP users in social media shows that HTP related content (e.g., photos of using the device) is often shared. This behavior pattern on social media promotes a community of people identifying with these products. All of these activities may increase the number of HTP users and, what is equally important, the social acceptability of tobacco use [18,19,20]. 

Governments should closely monitor the use of HTPs and consider how to regulate them as soon as possible, including smoke-free measures, restrictions on labelling, advertising, promotion, sponsorship and, finally taxation policies. These strategies are part of the MPOWER policy package ratified by the WHO. MPOWER stands for monitor tobacco use and prevention policies (M), protect people from tobacco smoke (P), offer help to quit tobacco use (O), warn about the dangers of tobacco (W), enforce bans on tobacco advertising, promotion and sponsorship (E), and raise taxes on tobacco products (R) [21]. Data from this monitoring are essential for a fight against a tobacco use epidemic to succeed. Only through accurate measurement can problems caused by tobacco use be understood and interventions be effectively managed and improved.

### 4.2. Frequency, Pattern of Use 

In 2019, over one fifth of Poles admitted to habitual (daily) cigarette smoking (21%) while among people aged 20–29 years it was 16.7%, according to the data presented by the Chief Sanitary Inspectorate in Poland [22]. Studies showed that men were more likely to be current cigarette smokers in national and international studies, similar to our study [1,11,22]. In this survey, men were more likely to be HTP users according to our research, however, in a national study, men and women were found to be HTP users with similar frequency [11,22]. The difference can be an effect of sample age ranges (18–33 vs. 15–60+) [11,22].

HTPs, as well as e-cigarettes, are relatively new forms of nicotine delivery [16]. According to the YUPESS study, the prevalence of e-cigarette smoking in university students from Central and Eastern Europe was 1.1% [3]. Growing popularity of HTPs confirms that the problem of HTPs is now as urgent and widespread as was the issue concerning e-cigarettes in the past few years. Moreover, our respondents were more likely to use HTPs (2.8%) than participants from a nationwide study (0.4%), where those two groups differed in age range. This could be a consequence of advertisements addressed to young people, promoting HTPs as a fashionable product. Moreover, in pursuance of the European Union (EU) directive, upon 20 May 2020, Member States shall prohibit the sale of combustible tobacco products containing flavorings in any of their components, but the directive does not include tobacco sticks (which are heated and not combustible products) [23]. Thus, people who are used to smoking menthol cigarettes, generally youths, will only have the option of menthol tobacco sticks. 

### 4.3. Safety and Opinions about HTPs

Heated tobacco products are promoted as an alternative to traditional cigarettes. On 25 January 2018, the Food and Drug Administration (FDA) Tobacco Products Scientific Advisory Committee voted not to accept claims that HTPs are less harmful than cigarettes (reduced risk product) [24]. However, two years later, in July 2020, the FDA did authorize them to be marketed with a reduced exposure statement [25]. Nevertheless, the WHO claimed that reducing exposure to harmful chemicals in HTPs does not render them harmless, nor does it translate in a reduced risk to human health [26]. Despite this, the advertisements may suggest that HTPs are safer and this may influence the opinion of regular and potential users. In addition, many studies which compare the consequences of using HTPs to traditional cigarettes are supported by tobacco manufacturers [27,28,29,30]. This fact permits the brand to set a course of the research and to persuade scientists.

In 2018, manufacturers of HTPs reported levels for 58 constituents in the aerosol, of which 40 are considered by the FDA as harmful or potentially harmful. All substances were lower in HTP emissions compared with the smoke of reference cigarettes [30,31]. However, an independent study examined another 53 harmful or potentially harmful components and the result was contrary, as the content was significantly higher in HTP emissions than in cigarette smoke [30]. 

The tobacco manufacturer’s advertisements of the harmfulness of heated tobacco products may mislead the public opinion [32]. In a study conducted in Japan, a group of 3600 cigarette and/or HTP smokers was asked about their perception of harmfulness of HTPs relative to cigarettes [33]. Almost half (47.5%) perceived that HTPs were less harmful than cigarettes [33]. In London, the motivation of HTPs users has been examined by a qualitative interview study. One of the main reasons for HTP use was because due to the belief that it is less harmful than combustible cigarettes [34]. The largest study in Europe including only HTP users was conducted in Switzerland using an online survey. In 89% of respondents, the HTPs were used because it was perceived to be less toxic than cigarettes [35]. According to our respondents, 95% believe that HTPs are not safe for health. 

Physicians’ behaviors toward smoking cessation and vaping cessation interventions are associated with physicians’ smoking status (*p* < 0.05) [36]. According to the WHO, any form of tobacco use is harmful to health. For this reason, it seems that doctors should be health ambassadors—and by giving up smoking, set an example for their patients. Moreover, new forms of nicotine-containing product are a challenge for pre- and postgraduate medical education. Medical education programs should include information on new forms of nicotine products (including e-cigarettes and HTPs). Due to the growing amount of scientific evidence on the health effects of e-cigarettes and HTPs use, postgraduate education is crucial to provide evidence-based smoking cessation counseling. Moreover, the policy and medical guidelines on e-cigarettes and HTPs use depend on the country’s anti-tobacco strategy (endgame or harm reduction). Currently, the endgame approach dominates in Poland. Further studies should evaluate the impact of HTPs marketing on physicians’ attitudes towards tobacco use.

This study had several potential limitations. In it we focused on the opinion of a specific group with a potentially broad knowledge about risks of introducing toxic substances into an organism. The study population is younger than the mean age of the Polish population, which is 39.7 years for men and 43 years for women [37]. Furthermore, the study population is completing a university education, which categorizes them into the potentially highly educated group. This means that the results cannot be considered as a popular public opinion in Poland and, thus, cannot be generalized. Thus, the findings cannot be compared to the group of general adults in Poland. However, the aim of the research was to assess frequency of HTP use among university students. Thus, our findings could be helpful to raise awareness about the prevalence of HTP among a sub-group of young adults. The authors are aware that there are different methods for assessing current smoking status. We adopted a very liberal determinant of classifying a student as current smoker/user. Also, if students completed the questionnaires together in class, their answers could be changed by consultation with their peers. The potential problem can be caused also by the definition of e-smoking session. We defined one session of e-cigarette as 15 puffs or approximately 10 min of use, as it has previously been suggested [38], but it may not have been understood by our respondents. Nonetheless, to the best of our knowledge, this study represents the latest update on the prevalence of past and current HTP use and perceived harmfulness among young adults. Even if the characteristics of the sample are very specific, we believe that the opinion of future young doctors is particularly important. Healthcare professionals are the most opinion-forming group in subjects connected to health. This public confidence can be used to increase the general awareness about the consequences of using tobacco products. In addition, young doctors or future doctors are more likely to use social media to communicate with their patients than their older colleagues, so they can influence the opinion of the society in a more direct way [39]. Our research may become the basis for further such research in Poland. Practical implications can result from using these data to shape the anti-smoking policy in Poland.

## 5. Conclusions

HTP usage is less popular than traditional smoking, but frequency of ever use indicates a growing interest in HTPs. Most medical students believe that HTPs are not safe for health, but attitudes toward HTPs differ depending on one’s smoking status. Awareness of HTPs presence concerned most of the students and this is mostly transmitted by family and friends. The range of marketing of HTPs is extremely significant, the ubiquitous advertisement such as stores and kiosks, as well as Internet advertising are noticeable by potential users, as confirmed by the present findings.

## Figures and Tables

**Table 1:** Difference between men and women according to their nicotine product use.

Type of Nicotine Containing Product Used	Total *n* = 1340		Women *n* = 838		Men *n* = 502		*p*
	*n*	% (95% CI) ^1^	*n*	% (95% CI) ^1^	*n*	% (95% CI) ^1^	
Traditional cigarettes (exclusive)	148	11.0 (9.4–12.9)	89	10.6 (8.6–12.9)	59	11.8 (9.1–14.9)	0.5 ^2^
E-cigarettes (exclusive)	30	2.2 (1.5–3.2)	13	1.6 (0.8–2.6)	17	3.4 (2.0–5.4)	0.03 ^2^
Dual users (traditional cigarettes + e-cigarettes or Heated Tobacco Products/Heated Tobacco Products + e-cigarettes)	27	2.0 (1.3–2.9)	12	1.4 (0.7–2.5)	15	3.0 (1.7–4.9)	0.05 ^2^
Heated tobacco products (exclusive)	19	1.4 (0.9–2.2)	9	1.1 (0.5–2.0)	10	2.0 (1.0–3.6)	0.2 ^3^
Non–user	1116	83.3 (81.2–85.2)	715	85.3 (82.7–87.7)	401	79.9 (76.1–83.3)	<0.01 ^2^

^1^ 95% CI—95% confidence interval ^2^ result of Chi-square test ^3^ result of Fisher Exact test.

**Table 2:** Pattern of smoking among exclusive nicotine containing product users.

**Duration of Use**	**Traditional Smokers**		**E-Cigarette Users**		**Heated Tobacco Product Users**	
	***n* = 139 ^2^**	**%(95% CI) ^1^**	***n* = 29 ^2^**	**%(95% CI) ^1^**	***n* = 17 ^2^**	**%(95% CI) ^1^**
Less than 6 months	32	23.0 (16.3–30.9)	11	37.9 (20.7–57.7)	9	52.9 (27.8–77.0)
6–12 months	14	10.1 (5.6–16.3)	4	13.8 (3.9–31.7)	4	23.5 (6.8–49.9)
1–5 years	81	58.3 (49.6–66.6)	13	44.8 (26.5–64.3)	4	23.5 (6.8–49.9)
More than 5 years	12	8.6 (4.5–14.6)	1	3.5 (0.1–17.8)	0	0.0 (0.0–19.5)
**Cigarettes/Tobacco Sticks/Sessions of E-Smoking Per Day**	**Traditional Smokers**		**E-Cigarette Users**		**Heated Tobacco Product Users**	
	***n* = 141 ^2^**	**%(95% CI) ^1^**	***n* = 27 ^2^**	**%(95% CI) ^1^**	***n* = 18 ^2^**	**%(95% CI) ^1^**
1–5	94	66.7 (58.2–74.4)	12	44.4 (25.5–64.7)	7	38.9 (17.3–64.3)
6–15	42	29.8 (22.4–38.1)	5	18.5 (6.3–38.1)	11	61.1 (35.8–82.7)
>15	5	3.6 (1.2–8.1)	10	37.0 (19.4–57.6)	0	0.0 (0.0–18.5)

^1^ 95% CI—95% confidence interval ^2^ the values differ due to the fact, that not every respondent answered the question.

**Table 3:** Opinions about HTP safety, safety for passive smokers and pregnant women, addiction to HTPs and usage in public spaces among medical students.

Perception of Heated Tobacco Products	Total		Heated Tobacco Product Users		Non-Heated Tobacco Product Users		*p*
**Heated Tobacco Products safe for health**	*n* = 1029 ^2^	% (95% CI) ^1^	*n* = 37 ^2^	% (95% CI) ^1^	*n* = 992 ^2^	% (95% CI) ^1^	
Yes	55	5.3 (4.1–6.9)	16	43.2 (27.1–60.5)	39	3.9 (2.8–5.3)	<0.01 ^3^
No	765	74.3 (71.6–77.0)	15	40.5 (24.8–57.9)	750	75.6 (72.8–78.3)	
No opinion	209	20.3 (17.9–22.9)	6	16.2 (6.2–32.0)	203	20.5 (18.0–23.1)	
**Possibility of becoming addicted to Heated Tobacco Products**	*n* = 1027 ^2^	% (95% CI) ^1^	*n* = 36 ^2^	% (95% CI) ^1^	*n* = 991 ^2^	% (95% CI) ^1^	
Yes	964	93.9 (92.2–95.3)	33	91.7 (77.5–98.3)	931	93.8 (92.1–95.2)	0.5 ^4^
No	9	0.9 (0.4–1.7)	2	5.6 (0.7–18.7)	7	0.7 (0.3–1.5)	
No opinion	54	5.3 (4.0–6.8)	1	2.8 (0.1–14.5)	53	5.4 (4.0–6.9)	
**Level of Heated Tobacco Product addiction**	*n* = 959 ^2^	% (95% CI) ^1^	*n* = 33 ^2^	% (95% CI) ^1^	*n* = 926 ^2^	% (95% CI) ^1^	
The same as traditional cigarettes	781	81.4 (78.8–83.9)	12	36.4 (20.4–54.9)	769	83.1 (80.5–85.4)	<0.01 ^4^
Lower than traditional cigarettes	93	9.7 (7.9–11.8)	14	42.4 (25.5–60.8)	79	8.5 (6.5–10.5)	
Higher than traditional cigarettes	85	8.9 (7.1–10.8)	7	21.2 (9.0–38.9)	78	8.4 (6.7–10.4)	
**Heated Tobacco Product usage in public spaces**	*n* = 1025 ^2^	% (95% CI) ^1^	*n* = 36 ^2^	% (95% CI) ^1^	*n* = 989 ^2^	% (95% CI) ^1^	
Allowed	266	26.0(23.3–28.8)	22	61.1 (43.5–76.9)	244	24.7 (22.0–27.5)	<0.01 ^4^
Prohibited	759	74.1(71.3–76.7)	14	38.9 (23.1–56.5)	745	75.3(72.5 –78.0)	
**Heated Tobacco Product safety for passive smokers’ health**	*n* = 1029 ^2^	% (95% CI) ^1^	*n* = 37 ^2^	% (95% CI) ^1^	*n* = 992 ^2^	% (95% CI) ^1^	
Yes	210	20.4 (17.9–23.0)	26	70.3 (53.0–84.1)	184	18.6(16.2–21.1)	<0.01 ^4^
No	546	53.1 (50.0–56.2)	6	16.2 (6.2–32.0)	540	54.4(51.3–57.6)	
No opinion	273	26.5 (23.9–29.3)	5	13.5 (4.5–28.8)	268	27.0(24.3–29.9)	
**Heated Tobacco Product safety for use by pregnant women**	*n* = 1030 ^2^	% (95% CI) ^1^	*n* = 37 ^2^	% (95% CI) ^1^	*n* = 993 ^2^	% (95% CI) ^1^	
Yes	8	0.8 (0.3–1.5)	2	5.4 (0.7–18.2)	6	0.6(0.2–1.3)	^5^
No	971	94.3 (92.7–95.6)	34	91.9 (78.1–98.3)	937	94.4(92.7–95.7)	
No opinion	51	5.0 (3.7–6.5)	1	2.7 (0.1–14.2)	50	5.0(3.8–6.6)	

^1^ 95% CI—95% confidence Interval. ^2^ the values differ due to the fact, that not every respondent answered the question. ^3^ result of Chi-square test. ^4^ result of Fisher Exact test. ^5^ not calculable due to small number of participants.

**Table 4:** Adjusted associations between personal characteristics and nicotine containing product use (*n* = 1344).

Variable	Ever			Current		
	Cigarettes OR (95% CI)	E-Cigarettes OR (95% CI)	HTP ^1^ OR (95% CI)	Cigarettes OR (95% CI)	E-Cigarettes OR (95% CI)	HTP OR (95% CI)
Sex						
Females	1.00	1.00	1.00	1.00	1.00	1.00
Males	1.0 (0.8–1.2)	1.4 (1.1–1.8)	1.3 (1.0–1.6)	1.3 (1.0–1.8)	2.5 (1.4–4.5)	2.00 (1.0–3.9)
Age	1.3 (1.1–1.4)	1.0 (0.9–1.1)	1.0 (1.0–1.1)	1.1 (1.0–1.3)	1.0 (0.8–1.3)	1.1 (0.9–1.4)
Year of studies	0.9 (0.8–1.0)	0.9 (0.8–1.00)	1.0 (0.9–1.1)	0.9 (0.8–1.0)	0.8 (0.6–1.1)	0.9 (0.6–1.2)

^1^*n* = 1342.

**Table 5:** Results (odds ratio) from the adjusted analysis looking at beliefs around health impacts among those who have heard of HTPs (*n* = 1030).

Statement	Males (Ref: Females)	HTPs Users (Ref: Non-User)	Age	Years of Study
	OR (95% CI)	OR (95% CI)	OR (95% CI)	OR (95% CI)
HTPs safe for health (ref: Yes) ^1^				
No	0.6 (0.4–1.2)	0.1 (0.0–0.1)	1.1 (0.9–1.3)	1.0 (0.7–1.3)
Don’t know	0.5 (0.3–1.0)	0.1 (0.0–0.2)	1.1 (0.8–1.3)	0.9 (0.7–1.3)
Can you become addicted to HTPs (ref: Yes) ^2^				
No	1.2 (0.3–4.5)	8.9 (1.8–45.8)	0.7 (0.3–1.4)	1.9 (0.8–4.6)
Don’t know	0.5 (0.3–0.9)	0.6 (0.1–4.4)	1.1 (0.9–1.3)	0.9 (0.7–1.2)
Should HTPs be banned in public places (ref: Yes) ^3^				
No	1.2 (0.9)	4.9 (2.5–9.8)	0.9 (0.8–1.1)	0.9 (0.7–1.0)
Are HTPs safe for passive smokers (ref: Yes)				
No	0.9 (0.7–1.3)	0.1 (0.0–0.2)	1.1 (1.0–1.3)	1.0 (0.8–1.2)
Don’t know	0.6 (0.4–0.9)	0.1 (0.1–0.4)	1.2 (1.0–1.3)	0.9 (0.7–1.1)
Are HTPs safe for pregnant women (ref: Yes)				
No	0.2 (0.0–1.1)	0.1 (0.0–0.6)	1.3 (0.7–2.5)	0.7 (0.3–1.6)
Don’t know	0.3 (0.1–1.6)	0.1 (0.0–0.8)	1.4 (0.7–2.8)	0.6 (0.3–1.6)

^1^*n* = 1029; ^2^ *n* = 1027; ^3^ *n* = 1025.

## Data Availability

Data are available from the corresponding author upon reasonable request.

## Supplementary Materials

The following are available online at https://www.mdpi.com/1660-4601/18/7/3381/s1, Study Questionnaire 1: Heated Tobacco Products Questionnaire.
