# Supplementary material for: The Frequency of Use and Harm Perception of Heated Tobacco Products (HTPs): The 2019 Cross-Sectional Survey among Medical Students from Poland
Source: Int J Environ Res Public Health. 2021 Mar 24;18(7):3381. doi: 10.3390/ijerph18073381 (PMC8037208; doi:10.3390/ijerph18073381)
Supplement: Supplementary file 1 [file ijerph-18-03381-s001.pdf]

# Heated Tobacco Products Questionnaire

**Dear Sir or Madam,**

Heated Tobacco Products (HTPs) as „IQOS” or „glo” are innovative tobacco products, which are advertised as alternative for traditional smoking. Those products, by heating dedicated tobacco sticks, lead to the production of a nicotine-containing aerosol. At present, the frequency of Heated Tobacco Products is not well known. We invite you to take part in a study on Heated Tobacco Products among students. The survey was prepared as part of a research project conducted by the Department of Epidemiology, SUM in Katowice.

1. Gender:

   [ ] Female [ ] Male

2. Age:

   ………………

3. Year of studies:

   ………………

4. Have you ever heard about heated tobacco products (e.g.: „IQOS”, „glo”)?

   [ ] Yes [ ] No

4.1 If "Yes", from which source have you heard about heated tobacco? (e.g.: „IQOS”, „glo”)? (please check all that apply)

[ ] Family/friends
[ ] Internet advertising
[ ] Television advertising
[ ] Scientific articles
[ ] Advertising materials - e.g. leaflets in packets of traditional cigarettes, billboards
[ ] Shop windows
[ ] Others: ………………………………

5. Have you ever smoked / tried a traditional tobacco cigarette?

[ ] Yes [ ] No

If "Yes", at what age did you smoke your first cigarette? ........... years

6. Have you ever smoked / tried an e-cigarette?

[ ] Yes [ ] No

If "Yes", at what age did you smoke your first e-cigarette? .......... years

7. Have you ever smoked / tried any of heated tobacco products (e.g.: „IQOS”, „glo”)?

[ ] Yes [ ] No

If "Yes", at what age did you use your first heated tobacco product? .......... years

7.1 If "Yes", under what circumstances did you use for the first time:

E-cigarette:

[ ] I have bought my own[ ] I was offered, for example, by a friend

Heated Tobacco Product:

[ ] I have bought my own [ ] I was offered, for example, by a friend

8. Do you currently smoke/use ……….? (please check all)

8.1 **Traditional cigarettes** [ ] Yes [ ] No

If "Yes", how long do you smoke traditional cigarettes?........... number of months

and how many cigarettes do you smoke on average per day ........... cigarettes / day

8.2 **E-cigarettes** [ ] Yes [ ] No

If "Yes", how long have you been using e-cigarettes..........number of months

and how many times per day do you use an e-cigarette..........number of sessions/day

8.3 **Heated Tobacco Products** (e.g.: „IQOS” or „glo”) [ ] Yes [ ] No

If "Yes", how long do you smoke HTPs?........... number of months

and how many tobacco sticks do you use on average per day.......... number of tobacco sticks/day

9. In the near future do you plan to start using Heated Tobacco Products?

[ ] Yes [ ] No [ ] Already using

10. Do you think Heated Tobacco Products are safe for your health?

[ ] Yes
[ ] No
[ ] No opinion

11. In your opinion, tobacco heating systems comparing to traditional cigarettes are:

[ ] Less harmful than traditional cigarettes
[ ] More harmful than traditional cigarettes
[ ] As harmful as traditional cigarettes
[ ] No opinion

12. In your opinion, tobacco heating systems comparing to electronic cigarettes are:

[ ] Less harmful than electronic cigarettes
[ ] More harmful than electronic cigarettes
[ ] As harmful as electronic cigarettes
[ ] No opinion

13. Do you think Heated Tobacco Products are safe for health of passive-smokers?

[ ] Yes
[ ] No
[ ] No opinion

14. Do you think Heated Tobacco Products are safe for using by pregnant women?

[ ] Yes
[ ] No
[ ] No opinion

15. Do you think you can become addicted to Heated Tobacco Products?

[ ] Yes

[ ] No

[ ] No opinion

15. 1 If "Yes", then:

[ ] HTPs are as addictive as a traditional cigarette

[ ] HTPs are less addictive than traditional cigarettes

[ ] HTPs are more addictive than traditional cigarettes

16. Have you ever encountered the form of promotion and marketing of heated tobacco ("IQOS" or "glo")? (questions with possible yes / no answer)

| | | |
|---|---|---|
| point-of-sale advertising - stores / kiosks etc. | [ ] Yes | [ ] No |
| Internet advertising | [ ] Yes | [ ] No |
| sale of heated tobacco at a promotional price | [ ] Yes | [ ] No |
| free trial at the point of sale | [ ] Yes | [ ] No |
| articles with the logo and name of heated tobacco (e.g. clothes) | [ ] Yes | [ ] No |
| sponsorship of cultural / sporting events by tobacco companies | [ ] Yes | [ ] No |
| booths of tobacco companies during mass events (e.g. concerts) | [ ] Yes | [ ] No |

17. In your opinion, according to the law in Poland, using Heated Tobacco Products in public places **is**:

[ ] permitted [ ] banned

18. In your opinion, do you think that using Heated Tobacco Products in public places should be banned?

[ ] Yes [ ] No

**Thank you for participating in the survey!**

In our study, we set the additional goal of knowing the immediate health effects of using tobacco heating systems. If you are currently using "IQOS" or "glo" and want to participate in the survey, please leave your email address and we will send you detailed information about the survey ……………………………….
